# Avian corticosteroid-binding globulin: biological function and regulatory mechanisms in physiological stress responses

**DOI:** 10.1186/s12983-021-00409-w

**Published:** 2021-04-29

**Authors:** Hai-Yan Lin, Gang Song, Fumin Lei, Dongming Li, Yanhua Qu

**Affiliations:** 1 Key Laboratory of Zoological Systematics and Evolution, Institute of Zoology, Chinese Academy of Sciences, Beijing, 100101 China; 2 Key Laboratory of Animal Physiology, Biochemistry and Molecular Biology of Hebei Province, College of Life Sciences, Hebei Normal University, Shijiazhuang, 050024 China

**Keywords:** Corticosteroid-binding globulin, Stress response, Glucocorticoids, Sex hormone-binding globulin

## Abstract

Corticosteroid-binding globulin (CBG) is a high-affinity plasma protein that binds glucocorticoids (GCs) and regulates their biological activities. The structural and functional properties of CBG are crucial to understanding the biological actions of GCs in mediating stress responses and the underlying mechanisms. In response to stress, avian CBGs modulate the free and bound fractions of plasma corticosterone (CORT, the main GC), enabling them to mediate the physiological and behavioral responses that are fundamental for balancing the trade-off of energetic investment in reproduction, immunity, growth, metabolism and survival, including adaptations to extreme high-elevation or high-latitude environments. Unlike other vertebrates, avian CBGs substitute for sex hormone-binding globulin (SHBG) in transporting androgens and regulating their bioavailability, since birds lack an *Shbg* gene. The three-dimensional structures of avian and mammalian CBGs are highly conserved, but the steroid-binding site topographies and their modes of binding steroids differ. Given that CBG serves as the primary transporter of both GCs and reproductive hormones in birds, we aim to review the biological properties of avian CBGs in the context of steroid hormone transportation, stress responses and adaptation to harsh environments, and to provide insight into evolutionary adaptations in CBG functions occurred to accommodate physiological and endocrine changes in birds compared with mammals.

## Corticosteroid-binding globulin (CBG) is a major determinant of glucocorticoid (GC) actions during stress

The stress response in vertebrates, including activation of the hypothalamic-pituitary-adrenal (HPA) axis and sympathetic adrenomedullary (SA) system, enables them to cope with various environmental perturbations and challenges, facilitating physiological and behavioral adaptations. The HPA axis is governed by the secretion of the corticotropin-releasing hormone (CRH) from the hypothalamus, which triggers the release of the adrenocorticotropic hormone (ACTH) from the anterior pituitary into the circulation. In turn, ACTH exerts its actions on the adrenal cortex and initiates the rapid release of glucocorticoids (GCs) into the blood. As the principal active forms of GCs, cortisol and corticosterone (CORT) are fundamental for orchestrating a series of behavioral and physiological activities in response to environmental challenges and restoring the homeostatic balance [1]. During acute stressful situations, rapid elevations in plasma GCs exert a plethora of central and systemic activities that are vital for promoting survival, such as acute enhancement of metabolic processes, inflammatory reaction, and energy mobilization. Subsequently, the negative feedback of GCs on the hypothalamus and pituitary effectively inhibits the adrenal secretion of GCs, allowing a return to behavioral and physiological homeostasis. On the other hand, chronic activation of the HPA axis and long-term elevated concentrations of GCs cause deleterious effects on reproduction, immunity, development, metabolic activities, behavior, etc., resulting in serious or even life-threatening sequelae [2].

The HPA axis is involved in many physiological functions making an organism’s response to environmental changes appropriate for its reproductive status. There exists a reciprocal relationship between the HPA and the hypothalamic-pituitary-gonadal (HPG) axes. Activation of the HPA axis and GC secretion, especially in response to extreme stressors, has an inhibitory effect on gonadal hormone secretion through central actions in the hypothalamus and pituitary, causing potential cessation of reproduction and facilitating survival under extreme circumstances [3, 4]. Alternatively, sex hormones such as testosterone and estrogen can modulate the response of the HPA axis by influencing the response and secretion of releasing factors and GCs [3, 5], thereby blunting stress responses to preserve reproduction.

In the systematic circulation, biologically active steroids are transported by steroid-binding proteins, including corticosteroid-binding globulin (CBG, also known as transcortin) and sex hormone-binding globulin (SHBG), with the former transporting GCs and progesterone, and the latter carrying androgens and estrogens. Mendel’s Free Hormone Hypothesis states that free hormone in the plasma is biologically active [6]; on the other hand, the protein-bound hormone concentration also affects intracellular hormone concentrations and its biologic activity [7]. Plasma CBG and SHBG bind steroids with high affinity and specificity, and play important roles in controlling steroid access to target tissues [8, 9].

The steroid-binding properties of CBG in the plasma have been determined in mammals and most non-mammalian terrestrial vertebrates, including amphibians, reptiles and birds [10]. In humans and rats, CBG binds as much as 90% of circulating cortisol or CORT [10, 11]. In addition to GCs, CBG also transports progesterone with a comparably high affinity [10, 12, 13]. The CBG protein shares little sequence homology with other steroid carriers, and belongs to the serine proteinase inhibitor A (SERPINA) superfamily, but lacks proteinase inhibitory properties [14]. Crystal structure investigations of rat or human CBG have revealed that it folds into a stressed SERPIN conformation when it binds GCs and progesterone, with the reactive center loop (RCL) fully exposed from the central β-sheet A; whereas it adopts a relaxed conformation when RCL undergoes proteolysis and inserts within the protein core, causing the irreversible release of its bound steroids [15–17]. In this allosteric mechanism that modulates steroid binding and release, helix D plays a key role via coupling RCL movement and the integrity of the steroid-binding site [18]. Therefore, the function of CBG in terms of its specific ligand binding and targeted steroid release links with the positioning of the RCL prior to and after protease cleavage [18]. Importantly, proteolytic cleavage of CBG results in a marked but not a complete loss of steroid binding activity, characterized by a ten-fold lower affinity. Thus circulatory GCs are buffered by two pools, intact CBG with a high affinity and to a far lesser extent by proteolytically cleaved CBG with a low affinity [19]. The latter pool, as a backup buffer to that of the intact CBG, is of physiological significance, particularly in inflammation and sepsis, representing a mechanism for the delivery and probably a direct release of the hormone to inflammatory loci [11]. Furthermore, plasma CBG is a glycoprotein, with 30% of its mass represented by N-linked oligosaccharide chain [14]. Mammalian CBGs have five to six N-glycosylation sites, one of which resides in RCLs in human and rat CBGs [20]. Glycosylation of CBGs influences steroid-binding activity, and disruption of a highly conserved N-glycosylation site causes a loss of steroid binding [20, 21].

It is therefore evident that CBG has functions beyond a simple plasma steroid transporter, and plays a major role in bioavailability, local delivery, and cellular signal transduction of GCs [22, 23]. This is evidenced in a mouse model genetically deficient for CBG, which exhibits fatigue, poor response to septic shock, and an inability to appropriately respond to excessive free CORT [22]. It has also been reported that CBG-deficient mice have markedly reduced total circulating CORT at rest, insufficient GC signaling, decreased endocrine (free CORT concentration) and behavioral responses after prolonged stress, as well as intolerance to life-threatening inflammation [24]. Interestingly, free CORT levels are normal under resting conditions in CBG-deficient mice [24], leading to the debate that the primary function of CBG seems to be the retention of a circulating pool readily available in an emergency situation [25]. In fact, the critical role of CBG as a cortisol reservoir, in particular for stress-induced CORT delivery to the brain, has been underlined by the following studies. CBG-deficient mice are insensitive to stress, have a blunted CORT response, no free CORT rise in the hippocampus and increased CORT clearance, stemming from a smaller CORT reservoir in blood [26, 27]). Remarkably, CBG is intrinsically expressed in various brain regions and in neurons and glial cells of humans and mice [28]. Thus, given that CORT unbound to CBG are cleared from the blood more quickly, the maintenance of a plasma pool of GCs by CBG should always be considered.

Patients with CBG deficiencies caused by nonsynonymous single nucleotide polymorphisms (SNPs) and amino acid substitutions often present with low plasma cortisol levels, are overweight, and suffer from chronic pain and/or fatigue. For example, homozygous or heterozygous carriers of CBG Lyon (D367N) and/or W12stop (W12 null mutation) have chronic asthenia, fatigue, hypotension, low morning total plasma cortisol levels, abnormal regulation of HPA axis and reduced cortisol peak levels upon ACTH stimulation [29, 30]. Mutations causing CBG deficiencies include transcortin Leuven (L93H) [31–34], Lyon (D367N) [29, 30, 35–37] and E102G [38] that have reduced steroid-binding affinity or capacity; G237V [39] and W371S [35] characterized by no detectable cortisol-binding activity; W12stop [30, 36] and L5stop [40] that are not produced due to null mutations; A51V [38, 41] that has reduced protein production and secretion. Most of these CBG deficiencies are considered rare, except for CBG A51V, which occurs at a relatively high frequency of approximately 1:36 in over 2000 Han Chinese [38]. Furthermore, the CBG rs7161521 SNP is also reported to be associated with diurnal and stress-induced salivary cortisol and HPA axis activity in children [42]. Thus, these mutations that influence CBG production or its cortisol-binding activity raised the functional importance of CBG. Free cortisol levels in individuals carrying D367N are within normal ranges [29], suggesting a net result or a homeostatic balance of reduced total cortisol level and appropriate response of the hypothalamic/pituitary adrenal axis.

Overall, mammalian CBGs have been characterized at the molecular level. SerpinA6 genes, which encode CBG, originated as a result of SerpinA gene duplications in early terrestrial vertebrate genomes [43]. Unlike mammalian CBGs, bird CBGs bind progesterone and androgen with high affinities and determine their biological activities [13, 44]. Moreover, accumulating evidence has documented the function of avian CBGs in endocrine and neural responses to stressors. The main goal of this review is to address the conserved/specialized functions of avian CBGs and to provide some insight into how they evolved to control steroid transport and bioavailability in response to environmental and physiological stressors.

## Coping with changing environments and adaptive stress responses in wild birds

When an unpredictable circumstance such as predation or adverse weather conditions occurs, a short-term stress response so called the “fight-or-flight” response is triggered within seconds to minutes in vertebrates. In birds, CORT is the primary GC involved in the modulation of stress response. In wild-caught birds subjected to a standardized capture-handling-restraint stress protocol, CORT levels usually increase within several minutes of initial capture and handling, then sustain until 30 to 60 min, and decline afterwards [45, 46]. Such an acute stress response is generally beneficial for immediate survival. However, if the stressor continues, CORT levels remain elevated and the bird enters long-term chronic stress response [47].

As volant vertebrates, birds have the widespread distribution range covering diverse environments globally, and display extraordinary diversities and plasticity in phenotypic traits and behavior, allowing them to adapt to the most extreme environments on the Earth, from the polar region to the Himalayan alpine. Under extreme conditions, birds have evolved various coping strategies of stress physiology, among which the dependence on extreme habitats is related to the balance between stress response patterns and reproductive requirements [48]. For instance, the Snow Petrel, *Pagodroma nivea*, is a long-lived bird with very low fecundity and often breeds in ice fields on or near the Antarctic continent. Young birds show increased CORT levels in response to acute stress and a tendency of nest abandonment, but this breeding disruption is also compromised in older individuals [48]. Indeed, it has been widely recognized that the GC response of arctic birds to unpredictable perturbation factors, such as inclement weather, patchy food and predators, is suppressed while breeding, allowing continued nesting and successful reproduction [49–52].

Over the last two centuries, urbanization, characterized by human population aggregation and urban expansion, has driven unprecedented environmental and ecological changes. Consequently, wild birds have had to face emerging stressors caused by profound changes in their habitats and food resources. A number of bird species that have adapted to urbanization are manifested by their strong dispersal abilities and a high level of risk-taking [53]. In a large-scale study of the juvenile House Sparrow (*Passer domesticus*) in urban and rural sites, feather CORT levels are positively correlated with the extent of urbanization and stress-induced plasma CORT levels, suggesting the impact of urban environmental conditions on stress physiology and sensitivity [54]. However, other studies show inconsistent profiles of stress physiology in different populations. The European Blackbird (*Turdus merula*) in urban areas have lower plasma CORT levels than their conspecifics living in forests [55]. The human commensal population of the House Sparrow shows lower stress-induced free CORT levels than its non-commensal population living in ancestral habitats [56]. In the urban populations of the Song Sparrow (*Melospiza melodia*) and the adult House Sparrow along the urbanization gradient, no detrimental effects of urbanization on stress physiology are observed [57, 58]. Notably, in the extreme environment of the Tibetan Plateau, there are no significant differences in acute adrenocortical responses in the Eurasian Tree Sparrow (*Passer montanus*) relative to the lowland populations, further suggesting a mask effect by human activities, food resources and shelter [46, 50]. Overall, different urban birds have different coping styles and urbanization-adapted stress physiology. The reduced stress response could be an adaptive strategy for species that adjust to and thrive in the urban environment, avoiding deleterious effects caused by chronic stress.

## The function of CBGs in avian stress responses

While modulating CORT synthesis/sensitivity of the HPA axis to feedback is one mechanism, altering CBG activity is another way for modulating the stress sensitivity to respond to environmental perturbations. In birds, CBG has been proved to function as a dynamic component and an essential mediator of the stress physiology in response to unpredictable perturbations. As aforementioned, free CORT is the biologically active fraction, while the CBG-bound fraction is a biologically relevant reservoir. Thus, the measurement of free GC concentrations in stress response has constraints and is not fully reliable [59]. In this respect, plasma levels of total CORT, free CORT and the binding affinity/capacity of CBG are all fundamental measurements in evaluative and comparative field studies to assess stress physiology of different bird species [59, 60]. For example, in the House Sparrow, CBG levels change correspondingly with total CORT levels, resulting in static free CORT concentrations year-round, whereas in the White-crowned Sparrow (*Zonotrichia leucophrys*), levels of CBG and free CORT change simultaneously when total CORT levels remain stable [61]. It is becoming clear that in response to stressors, changes in circulating GC levels and CBG capacity act in species-, life-history stage- and habitat environment-specific manners.

### Avian CBGs in species-specific stress responses

In response to the acute stress, the CBG binding capacity can also change when the total CORT increased significantly, which would result in remarkable variations in the circulating free CORT levels. Although the majority of bird species can remain CBG capacity invariable in response to acute handling stress, several species exhibit a decrease in the CBG binding capacities (reviewed by [62]). For example, several species such as the Common Tern (*Sterna hirundo*), the Red Crossbill (*Loxia curvirostra*), the Zebra Finch (*Taeniopygia guttata*), the American Kestrel (*Falco sparverius*) and the Laysan Albatross (*Phoebastria immutabilis*), CBG steroid-binding capacity significantly declines within 30–60 min of capture stress, serving to increase free CORT levels in response to acute stressors [62, 63]. Some other species remain static CBG capacities in response to the acute stress of capture-handling-restraint [63], e.g., the House Sparrow [63], the European Starlings (*Sturnus vulgaris*) [63], the Japanese Quail (*Coturnix japonica*) [63], the White-crowned Sparrow [63], the Black-legged Kittiwakes (*Rissa tridactyla*) [64], the Albert’s Towhee (*Pipilo aberti*) [65], the Canyon Towhee (*Pipilo fuscus*) [65], the Curve-billed Thrasher (*Toxostoma curvirostre*) [65] and the Northern Mockingbird (*Mimus polyglottos*) [65]. Interestingly, the CBG binding capacities of Eurasian tree sparrows can even increase in response to acute stress in the second nestling stage, although it remains stable in other life-history stages [66]. Furthermore, the CBG binding capacity also varies with nutrient status. In the White-crowned Sparrow, food deprivation at 1, 2, and 6 h significantly increases free CORT levels above baseline, whereas circulating levels of CBG are significantly reduced at 22-h fasting when free CORT reaches normal levels [67]. Such discrepancy of CBG changes in response to acute stress and nutrient status may be explained by some underlying protective mechanisms, such as the increase of bioavailable (free) CORT for minimizing extraordinary levels of CORT secretion, the regulation of negative feedback, the increase of glucose utilization, or upregulation of CORT-dependent metabolic functions (Reviewed by [59]). It is interesting that plasma CBG levels respond very dynamically in response to stress in some species but not in others, which suggests either rapid proteolysis of CBG or a marked increase in its plasma clearance. Considering that the causes and consequences of the variations in CBG binding capacity are rather complex that is involved in a suite of energy-dependent biochemical and molecular pathways [68, 69], further studies are needed to test the biological, ecological significance of CBG binding variations during the acute stress response or energy-dependent stress.

Apart from the binding capacity, the CBG binding affinity of birds also varies significantly with species (Reviewed by [62]), e.g., from 1.48 nM (Zebra Finch, *Taeniopygia guttata*) [63] to 25.4 nM (Pied Flycatcher, *Ficedula hypoleuca*) [70]). The observed species-specific CBG binding affinity could derive from the CBG protein topological structures. How the key amino acid sites evolve to increase the CBG binding affinity is critically essential for better understanding the interspecies differences of CBG binding affinity. Unfortunately, limited information is available for explaining this question to date. Further studies integrating the fields of biological function, phylogeny, and evolution are needed to uncover these unsolved questions.

### Life-history stage dependent variations of avian CBGs and stress responses

Birds have evolved a variety of life-history strategies in response to seasonality [71, 72]. Theoretically, animals with fewer reproductive opportunities are expected to invest more value over current reproduction. Birds with fewer breeding chances (short-lived within a lifetime or limited opportunities to re-nest within a season) should reduce stress reactivity to minimize the risk of nest abandonment [64, 73], then CORT and CBG levels change accordingly. In most avian species, seasonal fluctuations of baseline and stress-induced CBG capacity and CORT release reflect their life-history stage dependent strategies for adapting to environmental variations through optimizing their physiological and behavioral states. For example, in the House Sparrow, capture-handling-restraint stress responses during the pre-basic molt are lower than those during the breeding; in addition, the seasonal regulation of CORT response appears to be correlated with the HPA axis sensitivity that also varies seasonally [56, 74]. In the Eurasian Tree Sparrow, seasonal fluctuations of stress responses also show sex-specific patterns. Male birds have higher baseline CBG capacities during the nest-building, the first egg-laying, and the first nestling stages, and increased stress-induced CBG capacities during the second nestling stage [66]. Females have higher baseline plasma CBG levels during the nest-building stage, increased stress-induced CBG during the second egg-laying and the second nestling stages, but decreased stress-induced CBG during the nest-building stage [66]. Moreover, CORT response and CBG levels also vary with breeding sub-stages, i.e., maximal free CORT levels are lower during the nest-building stage than those during the early nestling stages, and females exhibit lower maximal CORT during the early nestling compared to later stages, suggesting the intensity of the adrenocortical response to acute restraint stress is negatively correlated with reproductive investment during breeding [75]. In a high-productivity breeding colony of the Tufted Puffins (*Fratercula cirrhata*), levels of CBG, total baseline CORT, free baseline CORT, and total maximum CORT are all higher during the pre-egg-laying stage than for the late incubation and late chick-rearing stages. Moreover, total baseline levels of CORT during the chick-rearing stage are 2–4 times higher at the low productivity colony, suggesting the higher cost of reproduction performance [76]. Overall, it is important to incorporate CBG and free CORT analysis into studies of the stress response, which would be better for explaining the relationship of stress reactivity to life-history strategies [62]. We may receive bias conclusions if solely relying on a value whereas in fact, CBG levels and free CORT may differ. Therefore, ignoring the effect of binding globulins may lead to a misinterpretation of the stress responses in birds [62].

### CBG and CORT levels coping with extreme environmental conditions - high latitudes

As mentioned above, avian species breeding in harsh environments, including high latitudes and elevations, have reduced adrenocortical responses to long-term environmental stress, enabling them to maximize reproductive success as a physiological trade-off [49, 51, 52]. The Arctic birds during breeding season, such as the Lapland Longspur (*Calcarius lapponicus*), the Common Redpoll (*Carduelis flammea*), the Snow Bunting (*Plectrophenax nivalis*) at Barrow and Toolik Lake, the Pied Flycatcher (*Ficedula hypoleuca*) and the Willow Warbler (*Phylloscopus trochilus*) breeding in Swedish Lapland (Ammarnäs), diminish the sensitivity of the adrenocortical response to acute stress as well as behavioral and physiological responses to CORT treatment, allowing birds to adapt to territorial behavior and to breed successfully in the face of the capricious environment [51, 77, 78]. In a comparative study of populations of the White-crowned Sparrow breeding at different latitudes, baseline and stress-induced CORT levels are similar; however, CBG binding capacity is significantly higher in subspecies *gamblii* breeding at high latitude than in *pugetensis* or *oriantha* breeding at middle or low latitude [79]. As a result, *gambelii*, with the shortest breeding seasons and the lowest free CORT levels among the three subspecies, is the least sensitive to environmental perturbations [79]. Arctic birds also suppress their adrenocortical response to acute capture-handling-restraint stress, especially in birds providing most parental care [80]. Besides, the suppression level varies with the intensity of the parental care. In the Rock Ptarmigan (*Lagopus mutus*), the Pectoral Sandpiper (*Calidris melanotos*) and the Red Phalarope (*Phalaropus fulicaria*), either females or males that provide most parental care, show lower stress-induced CORT levels and GC responses during the breeding season [80, 81].

### CBG and CORT levels coping with extreme environmental conditions - high elevations

In another highly extreme environment, the Tibetan Plateau, two endemic species, the White-rumped Snowfinch (*Onychostruthus taczanowskii)* and the Rufous-necked Snowfinch (*Pyrgilauda ruficollis)*, show no significant variation in adrenocortical response to stress between the early breeding and the pre-basic molt stages, which differs from those observed in arctic birds [49]. However, the extent of lowered adrenocortical responses can vary seasonally. The White-rumped and Rufous-necked Snowfinches have remarkably suppressed acute stress-induced CORT levels during the wintering stage relative to other stages, such as the early breeding, late breeding and pre-basic molting [82]. Moreover, the Twites (*Carduelis flavirostris*) on the Tibetan Plateau have lowered adrenocortical responses during pre-basic molt than those during the early breeding [78]. Overall, blunt adrenocortical responses, as physiological and ecological strategies, allow avian species to cope with extremes and to obtain maximal reproductive success through modulating the trade-off of energetic investment between reproduction and immediate survival. We predict that the higher binding capacity of CBG in extreme circumstances would contribute to reducing stress-induced free CORT levels for buffering the acute stress sensitivity.

## Zebra finch CBG crystal structure illustrates evolutionary conserved and distinct properties of avian CBGs

**Fig. 1:**
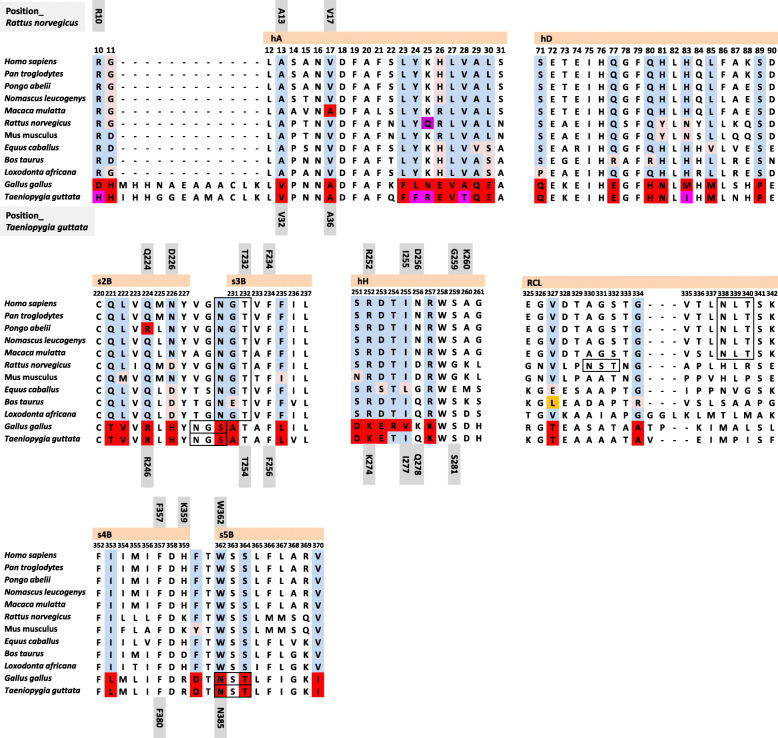
Multiple sequence alignment of CBG protein sequences from mammals, chicken and Zebra Finch are created in MEGA 7.0 [84] using the ClustalW algorithms. Regions involved in steroid binding and release, including helix A (hA), helix D (hD), helix H (hH), β-sheet B (s2B, s3B, s4B, s5B) and RCL [18, 83], are shown. Residue numbering is defined as those in rat CBG sequence [18]. Residues whose side chains directly interact with cortisol in the crystal structures of rat CBG and Zebra Finch CBG, are marked by grey boxes. Specific residues in chicken and/or Zebra Finch CBGs different from those of mammalian CBGs are highlighted in red or magenta boxes, with other residues in mammals marked by boxes in different light colors. Consensus sequences for N-linked glycosylation are shown in black boxes. *Homo sapiens*, human; *Pan troglodytes*, Chimpanzee; *Pongo abelii*, Sumatran Orangutan; *Nomascus leucogenys*, White-cheeked Gibbon; *Macaca mulatta*, Rhesus Macaque; *Rattus norvegicus*, Brown Rat; *Mus musculus*, mouse; *Equus caballus*, horse; *Bos taurus*, cow; *Loxodonta africana*, African Elephant; *Gallus gallus*, chicken; *Taeniopygia guttata*, Zebra Finch

Primary sequences of avian CBGs share limited identity with mammalian CBGs [83] (Fig. 1). Chicken (*Gallus gallus*) and Zebra Finch CBGs have been experimentally isolated and identified by mass spectrometry and molecular cloning, which provides evidence that chicken ENSGALG00000010969 and Zebra Finch ENSTGUG00000012647 (or LOC100228673) genes encoding CBG are incorrectly annotated as SerpinA4 and alpha-1-antiproteinase 2, respectively [83]. To the best of our knowledge, CBG in other avian species, as yet, has not been correctly annotated as CBG or SerpinA6. Notably, orthologs of CBG have been identified in several reptiles, whose CBG sequences are more similar to birds than mammals, especially within their steroid-binding sites, and their N-glycosylation and RCL sequences [83], indicating that SerpinA6 is an evolutionarily conserved gene.

The crystal structure of Zebra Finch CBG in complex with cortisol has been solved to 2.4 Å resolution [83]. The three-dimensional structures of Zebra Finch [83], rat [18] and human [85, 86] CBGs binding with cortisol resemble each other and exhibit evolutionarily conserved properties. However, their steroid-binding sites differ in several important aspects. Firstly, in mammalian CBGs, a conserved tryptophan residue (W362 in rat and W371 in human) in the steroid-binding pocket, which is critical for steroid-ligand binding, distinguishes it from other members of the SERPINA family; however, in Zebra Finch CBG, this tryptophan is replaced by an asparagine (N385), and this asparagine residue is present in this position in other avian CBG sequences. Secondly, among the 10–12 specific residues of rat CBG that directly participate in steroid binding, only 5 are conserved in Zebra Finch or other avian CBGs, and these differences account for the distinct high-affinity steroid-binding properties of avian CBGs for progesterone and androgens as well as GCs. Thirdly, mammalian CBGs have five or six N-glycosylation sites, some of which are strictly conserved and essential for steroid-binding activity [14, 20, 87]. By contrast, Zebra Finch CBG has only three N-glycosylation sites, one of which, N385, has been proved to participate in proper protein folding and high affinity steroid-binding site formation [83].

**Fig. 2:**
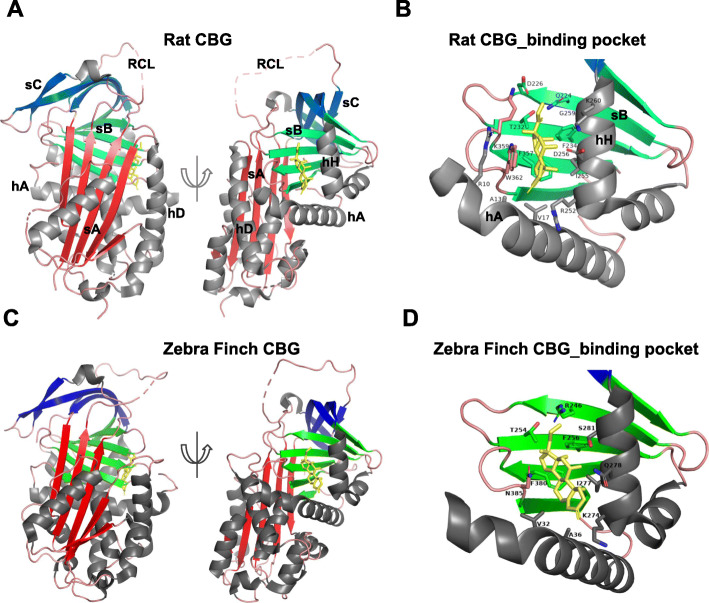
Crystal structures and binding pockets of rat CBG (PDB code: 2v95) [18] and Zebra Finch CBG (PDB code: 5hgc) [83]. A, the native rat CBG-cortisol structure. B, the cortisol-binding pocket of rat CBG. C, the native Zebra Finch CBG-cortisol structure. D, the cortisol-binding pocket of Zebra Finch CBG. The β-sheets A, B, and C are in red, green, and blue, respectively. Helixes are in grey. Cortisol is shown as stick models and coloured in yellow. The cartoons are prepared using PYMOL (http://pymol.sourceforge.net). In rat CBG, residues that directly interact with cortisol are R10, A13, V17 (in hA); Q224, D226 (in s2B); T232, F234 (in s3B); R252, I255, D256, G259, K260 (in hH); F357, K359 (in s4B); W362 (in s5B). In Zebra Finch CBG, cortisol-interacting residues include V32, A36 (in hA); R246 (in s2B); T254, F256 (in s3B); K274, I277, Q278, S281 (in hH); F380 (in s4B); N385 (in s5B)

The GCs in both Zebra Finch CBG and rat CBG crystal structures are at the interface of helix A, helix H and β-sheet B (Fig. 2). There are also important differences in the positioning of cortisol in the Zebra Finch CBG and rat CBG steroid-binding sites. Firstly, in rat CBG, the cortisol A ring forms a hydrophobic bond with A13 and V17, while in Zebra Finch CBG, it is held by V32 and A36. Secondly, in rat CBG, W362 forms a hydrogen bond and strong stacking interactions with the surface of cortisol, whereas in Zebra Finch CBG, N385 forms a hydrogen bond with the hydroxyl group of cortisol at C17 [83]. Overall, the Zebra Finch CBG structure reveals not only an evolutionarily conserved property but also the distinct steroid-binding activities of bird CBGs when compared with mammalian CBGs.

## Key characteristics of avian CBGs distinct from mammals: specific transporters of androgens

High affinity binding of dihydrotestosterone (DHT), testosterone and estradiol to SHBG have been observed in the blood of amphibians, reptiles and mammals [10]. Remarkably, in birds, plasma SHBG has never been identified [10, 13, 44] and gene encoding SHBG appears to be absent. Thus a specific transporter for androgens and estrogens in birds has remained elusive. In the Dark-eyed Junco (*Junco hyemalis*), CBG binds to CORT and progesterone with essentially similar high affinity, i.e., with the equilibrium dissociation constant for [^3^H] CORT of < 5 nM, whereas it binds androgens with approximately five-fold lower affinity [44]. However, this is still within the nanomolar range, and more than 90% of circulating testosterone is assumed to be bound with CBG in this species [44]. Comparable binding properties of CBG with CORT, progesterone and testosterone are also observed in 23 avian species belonging to 8 orders and 12 families [13]. A detailed study shows that Zebra Finch CBG has greater affinities for cortisol and progesterone (IC50 value of ~ 2 nM) than for CORT (~ 4 nM), and moderate affinities for testosterone (~ 18 nM) and DHT (~ 11 nM) [83]. Therefore, avian CBG is considered to substitute SHBG in transporting androgens and regulating their bioavailability [44].

In the crystal structure of Zebra Finch CBG, R246 forms a hydrogen bond with the hydroxyl group of cortisol at C21 and the carbonyl group at C20. Remarkably, substitution of R246 with glutamine significantly decreases the binding affinity for progesterone but does not affect affinities for cortisol and testosterone. Therefore, R246 might be the key residue that determines the higher affinity of avian CBGs for progesterone, but not androgens, than for mammalian CBGs [83]. Given the compensatory function of bird CBG in transporting androgen, key mechanisms that govern androgen binding remain to be addressed. Functional studies of distinct residues of Zebra Finch CBG, such as N385 and other residues in the steroid-binding pocket, can be performed via mutagenesis to answer whether they determine androgen-binding properties.

## Conclusions and perspectives

As reviewed above, CBG functions beyond a mere plasma carrier protein and regulates biologically active fractions of circulating steroid hormones, and is therefore considered as a primary gatekeeper of steroid actions [9]. Since in vitro experimental and in vivo evidence have proved a single amino acid substitution in key domains of both mammalian and avian CBGs has profound effects on steroid binding properties, manipulation of CBG function via in-depth molecular, cellular and genetic studies in birds would provide more biological and ecological perspectives on how the unique structure of avian CBG determines its specialized androgen binding function, and how GCs and sex hormones are regulated during development, breeding, physiological behavior and adaptation in birds.

Evolutionary analysis of the SERPIN superfamily, to which CBG belongs, has revealed the linkage between signatures of positive Darwinian selection and the molecular basis of adaptive evolution. Evidence of strong positive selection has been detected in SERPINB4 and B3, as important drivers of adaptive evolution of mammals [88, 89]. Specially, the RCL region, the critical determinant of target proteinase recognition, is hypervariable and attributed to accelerated rates of evolution that drives the functional specificities and diversification [89–92]. In this regard, integrated phylogenetic, evolutionary and topographic analysis of CBGs of multiple organisms over broad taxonomic groups, such as those of mammals and birds, will provide the bedrock for understanding evolutionary conservation and species-specific adaptations in the process of endocrine responses to both extreme environmental perturbations and stressors associated with human activities in the age of Anthropocene.

## Data Availability

Not applicable.

## Abbreviations

ACTH: Adrenocorticotropic hormone
CBG: Corticosteroid-binding globulin
CORT: Corticosterone
CRH: Corticotropin-releasing hormone
DHT: Dihydrotestosterone
GCs: Glucocorticoids
HPA: Hypothalamus-pituitary-adrenal
RCL: Reactive center loop
SERPINA: Serine proteinase inhibitor A
SHBG: Sex hormone-binding globulin
SNPs: Single nucleotide polymorphisms
